# A 64-week, multicenter, open-label study of aripiprazole effectiveness in the management of patients with schizophrenia or schizoaffective disorder in a general psychiatric outpatient setting

**DOI:** 10.1186/1744-859X-9-35

**Published:** 2010-09-17

**Authors:** Ming-Hong Hsieh, Wei-Wen Lin, Shao-Tsu Chen, Kao-Ching Chen, Kuang-Peng Chen, Nan-Ying Chiu, Chao Huang, Ching-Jui Chang, Cheng-Hsiu Lin, Te-Jen Lai

**Affiliations:** 1 Department of Psychiatry, Chung Shan Medical University Hospital and Institute of Medicine, Chung Shan Medical University, Taichung, Taiwan; 2 Department of Psychiatry, Tri-Service General Hospital, National Defense Medical Center, Taipei, Taiwan; 3 Peaceful Mind Psychiatry Clinic, Taoyuan, Taiwan; 4 Buddhist Tzu Chi General Hospital and University, Hualien, Taiwan; 5 National Cheng Kung University Hospital, Tainan, Taiwan; 6 National Taiwan University Hospital, Yun-Lin Branch, Yun-Lin, Taiwan; 7 Changhua Christian Hospital, Lu-Tung Branch, Changhua, Taiwan; 8 Wei Gong Memorial Hospital, Miaoli, Taiwan; 9 Cathay General Hospital, Taipei, Taiwan; 10 Catholic Mercy Hospital, Hsinchu, Taiwan

## Abstract

**Objective:**

To evaluate the overall long-term effectiveness of aripiprazole in patients with schizophrenia in a general psychiatric practice setting in Taiwan.

**Methods:**

This was a prospective, open-label, multicenter, post-market surveillance study in Taiwanese patients with a Diagnostic and Statistical Manual of Mental Disorders, Fourth Edition (DSM-IV) diagnosis of schizophrenia or schizoaffective disorder requiring a switch in antipsychotic medication because current medication was not well tolerated and/or clinical symptoms were not well controlled. Eligible patients were titrated to aripiprazole (5-30 mg/day) over a 12-week switching phase, during which their previous medication was discontinued. Patients could then enter a 52-week, long-term treatment phase. Aripiprazole was flexibly dosed (5-30 mg/day) at the discretion of the treating physicians. Efficacy was assessed using the Clinical Global Impression scale Improvement (CGI-I) score, the Clinical Global Impression scale Severity (CGI-S) score, The Brief Psychiatry Rating Scale (BPRS), and the Quality of Life (QOL) scale, as well as Preference of Medicine (POM) ratings by patients and caregivers. Safety and tolerability were also assessed.

**Results:**

A total of 245 patients were enrolled and switched from their prior antipsychotic medications, and 153 patients entered the 52-week extension phase. In all, 79 patients (32.2%) completed the study. At week 64, the mean CGI-I score was 3.10 and 64.6% of patients who showed response. Compared to baseline, scores of CGI-S, QOL, and BPRS after 64 weeks of treatment also showed significant improvements. At week 12, 65.4% of subjects and 58.9% of caregivers rated aripiprazole as better than the prestudy medication on the POM. The most frequently reported adverse events (AEs) were headache, auditory hallucinations and insomnia. A total of 13 patients (5.3%) discontinued treatment due to AEs. No statistically significant changes were noted with respect to fasting plasma glucose, lipid profile, body weight, and body mass index after long-term treatment with aripiprazole.

**Conclusions:**

Although the discontinuation rate was high, aripiprazole was found to be effective, safe and well tolerated in the long-term treatment of Taiwanese patients with schizophrenia who continued to receive treatment for 64 weeks.

## Background

Randomized, placebo-controlled studies using strict inclusion and exclusion criteria are essential when evaluating the efficacy and safety of a new treatment; however, they do not provide a measure of overall effectiveness (that is, whether or not the treatment works in real-world clinical practice) [1,2]. Effectiveness is a more global measure that considers not only efficacy and tolerability but also broader issues related to everyday clinical care such as patient preference [2]. Effectiveness studies are especially relevant where there may be differences in the standard of care or treatment practices, such as in countries where differences in healthcare systems and cultural preferences may further impact on treatment effectiveness.

Antipsychotic drugs are the primary treatment option for schizophrenia [3] and are increasingly studied in real-world settings. Although typical antipsychotic agents have a long history of use and are still used extensively in some parts of the world, atypical antipsychotics provide some advantages [3]. Atypical agents are effective in reducing both the positive and negative symptoms of schizophrenia and are also associated with a lower incidence of extrapyramidal symptoms and hyperprolactinaemia, both side effects that are commonly associated with typical antipsychotic agents [3].

Aripiprazole is a novel atypical antipsychotic with potent partial dopamine receptor D_2 _and D_3 _agonist activity [4,5], serotonergic 5-hydroxytryptamine (5-HT)_2A _antagonist activity [6] and 5-HT_1A _partial agonist activity [7,8]. In addition, aripiprazole has minimal affinity for α2 adrenergic receptors, H_1 _histamine receptors and muscarinic cholinergic receptors [9,10], possibly underlying the diminished liability of aripiprazole to produce orthostatic hypotension, sedation and weight gain, and cognitive impairment, respectively.

Aripiprazole has been shown to be efficacious and well tolerated for the treatment of schizophrenia in short-term (4-6 weeks) [11-15] and longer-term (26 and 52 weeks) clinical trials [9,12,16,17]. However, whether these findings will translate into long-term improvements in real-life clinical practice in a Taiwanese population warrants further study. Here, we report the results of a multicenter, open-label, post-market surveillance study to evaluate the overall long-term effectiveness of aripiprazole in Taiwanese patients with schizophrenia or schizoaffective disorder in a general psychiatric practice setting. The effectiveness of aripiprazole was evaluated in patients switching from other antipsychotic agents and the study was designed to reflect general psychiatric treatment practice.

## Methods

### Study design

This was a prospective, open-label, multicenter, post-market surveillance study to evaluate the long-term efficacy and safety of aripiprazole for the treatment of schizophrenia. The study took place between August 2006 and December 2008 at nine centers under the latest applicable International Conference on Harmonisation of Technical Requirements for Registration of Pharmaceuticals for Human Use (ICH) Good Clinical Practice (GCP), Guidelines of Taiwan and in accordance with the Declaration of Helsinki. The study protocol was approved by an Institutional Review Board committee from the nine medical centers, and all patients were required to provide written informed consent to participate. The study consisted of two phases: a 12-week switching phase during which time eligible subjects were switched from their current treatment to aripiprazole, followed by a 52-week long-term treatment phase during which subjects continued aripiprazole treatment for an additional 52 weeks.

### Subjects

This study enrolled male and female subjects, aged 18-65 years, with a Diagnostic and Statistical Manual of Mental Disorders, Fourth Edition (DSM-IV) diagnosis of schizophrenia or schizoaffective disorder. Patients were required to be currently taking antipsychotic drugs but warranting a change of antipsychotic medication because medication was not well tolerated and/or clinical symptoms were not well controlled, based on the clinical judgment of the investigator.

Patients were excluded from the study if they had acute psychosis, acute suicidal ideation, any acute psychiatric condition that might require emergent intervention, organic, neurological, cardiovascular diseases, or if they had a history of drug or alcohol abuse within the last 12 weeks. Other exclusion criteria were pregnant or breastfeeding women, or those planning a pregnancy during the study period; those receiving electroconvulsive therapy within 4 weeks prior to the screening visit; and those with a known allergic reaction to any antipsychotic medication (including but not limited to haloperidol, chlorpromazine, thioridazine, pimozide, risperidone, quetiapine and ziprasidone). Subjects with any clinical condition or significant concurrent disease judged by the investigator to complicate the evaluation of the study treatment were also excluded, as were those having participated in another study or taken the investigation drug within 1 month prior to study entry. Depot neuroleptics were discontinued at least 2 months prior to enrollment.

### Study treatments

Eligible subjects received their first dose of aripiprazole at the start of the switching treatment period, the starting dose of which was determined by the investigator. After 2 weeks of adverse event (AE)-free aripiprazole dosing, the treating physicians could increase the dose of aripiprazole to a maximum of 30 mg/day. Dosing could be reduced at any time due to tolerability. During the switching period (weeks 8-12), all prior antipsychotic medications were gradually discontinued; treatment with antipsychotics other than aripiprazole was not permitted during the 52-week long-term treatment phase. Treatment compliance was measured as the percentage of the actual days of drug dispensed divided by the expected days of drug dispensed based on the assessment schedule. Patients with values between 80% and 100% were considered to be treatment compliant. Of the 153 patients enrolled in the study, 106 patients (69.2%) were compliant with treatment.

Concomitant treatment with lorazepam and/or diphenhydramine was permitted during switching for the treatment of AEs but was not permitted during long-term treatment. Additionally, psychotrophic drugs other than antipsychotics were permitted throughout the study based on clinical judgment.

### Study assessments

Subjects were evaluated at baseline (week 0), weeks 2, 8 and 12 of the screening phase and 24, 38, 51 and 64 of the long-term treatment phase. During screening, additional visits could also be conducted at weeks 4 and 6 based on the chosen titration schedule of the investigator.

Effectiveness was evaluated using the Clinical Global Impression scale Improvement (CGI-I) score [18], the mean change from baseline in Clinical Global Impression scale Severity of Illness (CGI-S) score, the total score on the Brief Psychiatric Rating Scale (BPRS) [19], and the total score on the World Health Organization Quality of Life instrument, short version (WHOQOL-BREF) [20] questionnaire. Additional effectiveness measures included the CGI response rate (defined as a CGI-I score of 1 (very much improved), 2 (much improved) or 3 (minimally improved)) and the Preference of Medicine (POM) questionnaire completed during the 12-week switching phase by patients and caregivers [21]. All rating scales were completed by the treating physicians.

Safety measures included frequency and severity of AEs, serious AEs (SAEs); discontinuation due to AEs, abnormal laboratory results, physical examination, vital signs, body mass index (BMI), waist/hip measurement and metabolic syndrome parameters.

### Statistical procedures

The safety population was defined as all patients who received at least one dose of study medication, whereas the effectiveness population included all subjects who took at least one dose of study medication and had at least one post-baseline effectiveness measure. Continuous variables are presented as mean, standard deviation, median, range, and 95% confidence intervals (CIs). Paired t-tests were performed to compare baseline versus post-treatment. Categorical variables are presented in frequency tables. All analyses were performed on the observed case data set for each study week using the last observation carried forward (LOCF) method to handle missing data at study endpoint.

### Clinical trials registry

This study is registered with clinicaltrials.gov under the accession number NCT00520650.

## Results

### Subject demographics

**Figure 1:**
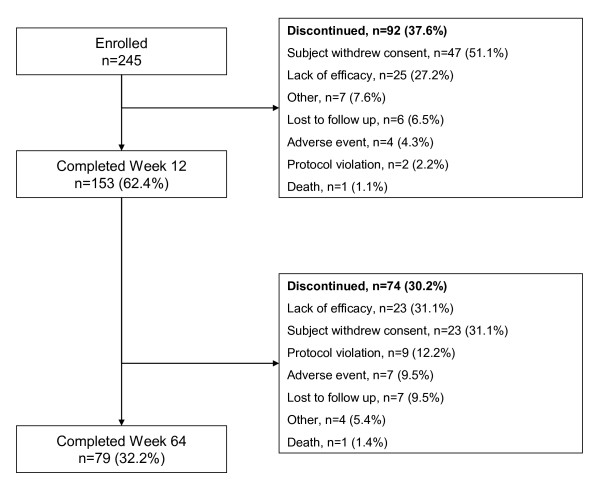
**Patient disposition**.

A total of 245 subjects were enrolled in this study, of whom 153 completed the 12-week switching phase and entered long-term treatment. Overall, 79 subjects (32.2%) completed the study. Subject disposition is shown in Figure 1. Subject baseline demographic characteristics are shown in Table 1.

**Table 1:** Baseline demographics characteristics (safety sample)

N	Aripiprazole (n = 245)
Gender, n (% male)	100 (40.8)
Mean ± SD age, years	37.0 ± 10.9
Schizophrenia, n (%)	244 (99.6)
Social history:	
Positive smoking history, n (%)	232 (94.7)
Alcohol or drug abuse within last 12 weeks, n (%)	0
CGI-S	3.84 ± 1.07

CGI-S = Clinical Global Impression scale Severity of Illness score.

### Study treatments

Antipsychotics received during switching included chlorpromazine (n = 2), clothiapine (n = 2), flupenthixol (n = 2), loxapine succinate (n = 1), sulpiride (n = 13), trifluoperazine (n = 1), haloperidol (n = 27), amisulpride (n = 14), risperidone (n = 89), ziprasidone (n = 13), zotepine (n = 14), clozapine (n = 5), olanzapine (n = 40) and quetiapine (n = 15). The mean ± SD aripiprazole daily dosage at the start of treatment was 8.93 ± 3.60 mg/day and 13.86 ± 6.46 mg/day at the final treatment (week 51).

During the first week of treatment, 29.0% of subjects were receiving aripiprazole at doses of ≤5 mg/day; 67.4% were receiving >5 mg/day and ≤15 mg/day, 3.3% were receiving >15 mg/day and ≤20 mg/day, and 0.4% were receiving >20 mg/day. At week 51 (n = 81), 9.9% of subjects were receiving aripiprazole at doses of ≤5 mg/day; 61.7% were receiving >5 mg/day and ≤15 mg/day, 19.8% were receiving >15 mg/day and ≤20 mg/day, and 8.6% were receiving >20 mg/day. Of the 153 patients enrolled in the study, 106 patients (69.2%) were compliant with treatment.

During the 12-week switching period, no patients received diphenhydramine and five patients received lorazepam for the treatment of restlessness.

### Efficacy

**Figure 2:**
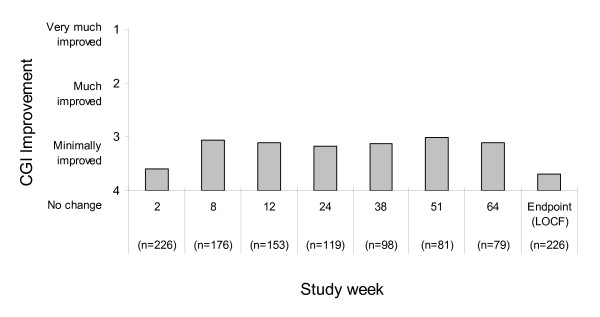
**Mean CGI-I score by study week (effectiveness sample)**. CGI-I = Clinical Global Impression scale Improvement score; LOCF = last observation carried forward.

**Figure 3:**
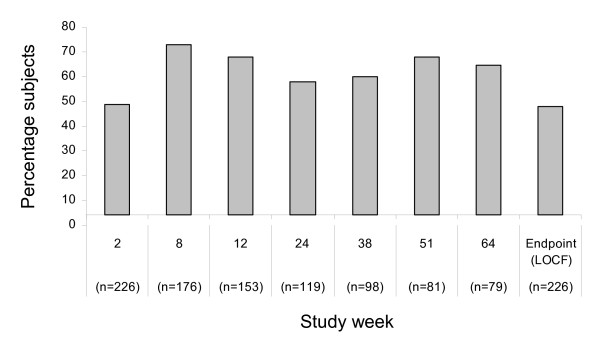
**Response rate by study week**. Response = CGI-I score of 1 (very much improved), 2 (much improved) and 3 (minimally improved). CGI-I = Clinical Global Impression scale Improvement score.

Mean CGI-I scores over the course of the study are shown in Figure 2. Mean CGI-I scores showed improvement during the course of the study; at week 64, the mean CGI-I score was 3.10 (95% CI 2.9 to 3.3). At endpoint (LOCF), the mean CGI-I score was 3.69 (95% CI 3.5 to 3.9). Figure 3 shows the percentage of subjects with a response (CGI-I score of very much or much improved or minimally improved) to treatment by study week. Response rates with aripiprazole were 64.6% and 47.8% at week 64 and study endpoint (LOCF), respectively.

Mean changes from baseline in QOL, CGI-S and BPRS ratings are shown in Table 2. Significant improvements were observed in scores of QOL, CGI-S and BPRS over the course of treatment compared to baseline (3.6 ± 12.0, *P *< 0.05; -0.7 ± 1.1, *P *< 0.001; -9.3 ± 11.6, *P *< 0.001, respectively).

**Table 2:** Mean change from baseline in additional effectiveness outcomes (effectiveness sample)

Outcome	Mean ± SD
WHOQOL-BREF:	
Baseline	71.0 ± 14.1
Change from baseline to week 12	2.2 ± 12.5*
Change from baseline to week 64	3.6 ± 12.0*
Change from baseline to endpoint (LOCF)	2.2 ± 11.3**
CGI-S:	
Baseline	3.8 ± 1.1
Change from baseline to week 12	-0.6 ± 1.0***
Change from baseline to week 64	-0.7 ± 1.1***
Change from baseline to endpoint (LOCF)	-0.3 ± 1.1***
BPRS:	
Baseline	49.8 ± 17.5
Change from baseline to week 12	-6.5 ± 10.5***
Change from baseline to week 64	-9.3 ± 11.6***
Change from baseline to endpoint (LOCF)	-5.8 ± 15.9***

*P < 0.05 vs baseline; **P < 0.01 vs baseline; ***P < 0.001 vs baseline.

BPRS = Brief Psychiatric Rating Scale; CGI-S = Clinical Global Impression scale Severity of Illness score; LOCF = last observation carried forward; WHOQOL-BREF = World Health Organization Quality of Life instrument, short version.

### Preference of medicine ratings

**Figure 4:**
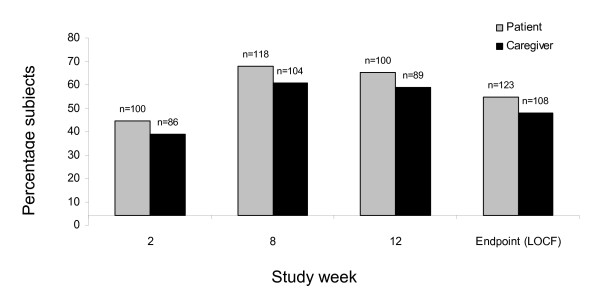
**Percentage of patients and their caregivers rating study medication as better than the previous antipsychotic medication during the 12-week switching period**. Subjects with a preference of medication rating of 'much better' or 'slightly better' were included in the total.

The percentage of patients and their caregivers rating study medication as 'much better' or 'slightly better' than their previous antipsychotic medication during the 12-week switching period is shown in Figure 4. Both patient and caregiver POM ratings for aripiprazole increased during the 12-week switching phase. At the end of the switching phase (LOCF), 54.7% of 123 subjects and 48.0% of 108 caregivers rated aripiprazole as 'slightly better' or 'much better' than the prestudy medication. For patients completing 12 weeks of treatment, 65.4% of subjects and 58.9% of caregivers rated aripiprazole as 'slightly better' or 'much better' than the prestudy medication.

### Safety and tolerability

Of the 245 subjects who were included in the safety sample, 163 (66.5%) experienced an AE during the switching treatment phase and, of the 153 subjects who entered long-term treatment, 110 (71.9%) of subjects experienced an AE. AEs that occurred in ≥5% of subjects are shown in Table 3. The most frequently reported AEs (occurring in ≥10% of subjects) during the entire study were headache, auditory hallucinations and insomnia. Most AEs were mild to moderate in severity.

**Table 3:** Adverse events occurring at an incidence ≥5%

Adverse event	n (%)
Headache	28 (11.4)
Hallucination, auditory	26 (10.6)
Insomnia	26 (10.6)
Constipation	23 (9.4)
Akathisia	14 (5.7)
Extrapyramidal disorder	13 (5.3)
Upper respiratory tract infection	13 (5.3)
Tremor	13 (5.3)

Discontinuations due to AEs occurred in 13 (5.3%) subjects. Adverse events leading to discontinuation included vision blurred, abdominal distension, dry mouth, nausea, irritability, dizziness, headache, syncope, aggression, agitation, disorientation, impulse control disorder, insomnia, persecutory delusion, psychotic disorder, restlessness, suspiciousness and thinking abnormal (all n = 1) and auditory hallucinations (n = 2); subjects may have discontinued treatment due to more than one AE. In addition, two subjects committed suicide during the study (one during switching and one during long-term treatment); both events were judged as being possibly related to study medication.

Serious AEs (SAEs) were reported in 43 (17.6%) subjects; of these, 9 were judged as being unlikely to be related to study drug, 14 as being unrelated to study drug, 11 as being possibly related to study drug, and 9 as being probably related to study medication by the investigators.

No clinically relevant changes in vital signs or other laboratory findings were observed over the course of treatment.

### Body weight and metabolic abnormalities

The mean ± SD weight was 67.3 ± 15.7 kg at baseline. For subjects continuing to receive treatment for 64 weeks, the mean weight increased over the course of treatment (68.9 ± 15.4 kg at week 64). The mean weight at study endpoint (LOCF) decreased (66.8 ± 15.1 kg). Neither of these changes from baseline were statistically significant. Mean ± SD BMI was 25.4 ± 5.2 kg/m^2 ^at baseline and was not significantly different at week 64 (25.8 ± 4.9 kg/m^2^; *P *= 0.719).

No statistically significant changes from baseline were noted for waist circumference, BMI, blood pressure, or high-density lipoprotein (HDL) levels. There was a significant reduction in the mean ± SD total cholesterol (baseline 185.6 ± 46.9 vs week 64 179.3 ± 38.8; *P *< 0.05), triglycerides (baseline 144.8 ± 150.7 vs week 64 120.0 ± 93.6; *P *< 0.001) and fasting plasma glucose (baseline 101.3 ± 39.1 vs week 64 96.7 ± 33.3; *P *< 0.05) over the course of aripiprazole treatment. There was a shift in glucose status over the course of treatment towards improvement; at baseline, 89.8% of subjects had normal blood glucose levels, whereas at week 64 and endpoint, 97.5% and 90.4% of subjects had normal levels, respectively.

### Prolactin

Aripiprazole treatment was associated with a significant decrease in serum prolactin levels over the course of treatment. At baseline, the mean ± SD serum prolactin level was 55.3 ± 60.9 ng/mL and at week 64 the mean ± SD prolactin level was 6.14 ± 5.46 ng/mL (*P *< 0.001). Only one subject reported hyperprolactinaemia as an AE.

## Discussion

Aripiprazole has previously been shown to be efficacious, safe, and well tolerated in randomized, placebo-controlled and active-controlled clinical trials [11,12,16,17]. This prospective, open-label, naturalistic study was designed to specifically evaluate the effectiveness of aripiprazole in a general outpatient psychiatric practice setting and to evaluate the effectiveness of aripiprazole in a Taiwanese patient population. Findings reported here demonstrate the broad effectiveness of aripiprazole in Taiwanese patients with schizophrenia or schizoaffective disorder and suggest that treatment is effective for up to 1 year of treatment. Mean CGI-I scores showed improvement over the course of both short-term and long-term treatment and the distribution of CGI-I scores at week 64 suggests that aripiprazole improved symptoms in a large proportion of patients; 64.6% of subjects had a clinically relevant improvement in symptoms at week 64, as measured by improvement in CGI-I scores. Furthermore, just over half of subjects who completed the switching treatment phase went on to receive 52 weeks of long-term treatment. Importantly, improvements in symptoms of schizophrenia were not limited to clinician assessment of symptoms; improvement in quality of life measured using the WHOQOL-BREF was also seen over the both short-term and long-term treatment period.

Results from this study suggest that patients requiring a switch in antipsychotic medication can benefit from switching to aripiprazole treatment in both the short and long term. In this study, patients and their caregivers generally preferred aripiprazole to their previous medication(s), as assessed using the preference of medication scale scores during the switching treatment phase. This finding, coupled with the improvement in QOL ratings, is important as it provides an estimation of the real-world impact of antipsychotic treatment with aripiprazole. As poor compliance is a primary reason for relapse [22], improvements in preference of medication (patient and caregiver) and QOL outcomes cannot be undervalued. However, longer-term studies using functional and social outcomes are needed to assess whether POM and QOL translate into meaningful changes in the long-term naturalistic treatment setting, such as improving the ability to return to work, reintegration with family, friends and improved social relatedness.

Although discontinuation rates over the 64 weeks of treatment were high, they are comparable to those reported with aripiprazole in other studies of similar duration [16,23] and high attrition rates are not uncommon in long-term trials [1]. It is, however, interesting to note that withdrawal of consent was the primary reason for study discontinuation during both the switching and long-term treatment phases. Although the most common reason for withdrawal of consent resulted from changes in personal circumstances, whether this was indirectly related to aripiprazole treatment is unknown.

The findings reported here in the Taiwanese population here are in agreement with previous prospective 8-week effectiveness trials of aripiprazole in patients with schizophrenia conducted in the US [21] and Europe [24]. These studies evaluated the broad effectiveness of aripiprazole in patients with schizophrenia and also reported a high proportion of patients preferring aripiprazole treatment over their previous medication when switching medication [21,24]. The findings reported here also agree with those of the Schizophrenia Trial of Aripiprazole (STAR) study, which demonstrated the longer-term effectiveness of aripiprazole for the treatment of schizophrenia in Europe [25]. This study demonstrated the effectiveness of aripiprazole compared with standard-of-care treatment in outpatient-treated patients with schizophrenia, especially in the areas of symptom improvement, clinical response, patient medication preference, and quality of life. The effectiveness of aripiprazole was also supported on a number of physical health dimensions [25].

Aripiprazole was generally well tolerated as a long-term treatment in this population, most AEs were mild to moderate in severity and discontinuations due to AEs were low. Furthermore, AEs reported here were consistent with findings from both long-term, randomized, placebo-controlled trials of treatment with aripiprazole in patients with schizophrenia [17] and findings from other long-term naturalistic studies [24]. It should be noted that, although two subjects committed suicide during this study, these events were only possibly related to study medication.

The metabolic side effects of medication, such as obesity, dyslipidemia, hyperglycemia and glucose intolerance, are an important consideration with long-term treatment due to their association with cardiovascular disease [26]. Furthermore, the atypical antipsychotics have been shown to differ significantly with respect to their effect on weight gain, plasma lipid levels, and insulin resistance [26,27]. Long-term aripiprazole treatment was not associated with a worsening of metabolic parameters in this study and, indeed, resulted in improvements in cholesterol, triglyceride and fasting plasma glucose levels at study endpoint compared to baseline. It should also be noted that there was a shift in glucose status over the course of treatment, with more patients having normal blood glucose levels at study end compared to baseline. Improvements in metabolic parameters following switching to aripiprazole has previously been observed [28] and suggests that patients developing metabolic abnormalities while receiving treatment with other antipsychotic agents may benefit from switching to aripiprazole.

Hyperprolactinemia, which is associated with some antipsychotic treatments, has a number of potential adverse clinical consequences, such as galactorrhea and gynecomastia, or endocrine-related secondary effects, such as sexual and reproductive dysfunction [29]. In the current study, long-term aripiprazole treatment was not associated with hyperprolactinaemia, but conversely a lowering of serum prolactin levels. This phenomenon can be explained by the pharmacodynamic mechanism of aripiprazole; partial agonist activity at D_2 _receptors. This does not lead to complete blockade of D_2 _receptors in the tuberoinfundibular tract, which is known to cause hyperprolactinaemia. Risperidone has also been studied as a long-term treatment in Taiwanese patients [30]. In this study, both oral risperidone and risperidone long-acting injection were shown to be effective and well tolerated over 48 weeks of treatment. Although oral risperidone increases serum prolactin levels, risperidone long-acting injection decreased serum prolactin levels over the course of treatment.

This study used a naturalistic study design that included a number of features designed to closely reflect clinical practice, such as the use of flexible study dosing and titration schedules based on clinical judgment and the use of relatively lenient inclusion and exclusion criteria compared to trials conducted for regulatory approval. However, it should be noted that the study did exclude subjects with current substance abuse or several somatic medical conditions. This may limit the generalizability of the study's findings as the population included in this study may not be fully representative of all patient types encountered in real-world settings. Furthermore, the findings reported here should also be interpreted within the context of the open-label study design. Additional limitations include the lack of comprehensive assessment on symptom improvement and the relatively high rate of study discontinuation. Finally, as this was not a controlled study, conclusions regarding the relative efficacy of aripiprazole compared with other antipsychotics in this Taiwanese population cannot be made.

## Conclusions

This is one of the longest studies conducted in a Taiwanese patient population and provides valuable information on the use of aripiprazole for the treatment of schizophrenia in this patient group, despite the relatively high discontinuation rates. Aripiprazole resulted in clinically meaningful improvement in the symptoms of schizophrenia that were evident throughout the 64 weeks of study treatment. Importantly, aripiprazole was well tolerated, supporting the broad effectiveness of aripiprazole for the treatment of schizophrenia in this patient group.

## Competing interests

The authors received research support from Taiwan Otsuka Pharmaceutical. The authors declare that they have no other competing interests.

## Authors' contributions

All authors contributed to the conception and design of study. WWL drafted the protocol. All authors contributed to finalize the protocol. All authors collected the study data. MHH, WWL and TJL reviewed and revised the manuscript. MHH and WWL read and approved the final manuscript.
